# Cigarette smoking worsens systemic inflammation in persons with metabolic syndrome

**DOI:** 10.1186/1758-5996-6-79

**Published:** 2014-07-16

**Authors:** Omar Jamal, Ehimen C Aneni, Sameer Shaharyar, Shozab S Ali, Don Parris, John W McEvoy, Emir Veledar, Michael J Blaha, Roger S Blumenthal, Arthur S Agatston, Raquel D Conceição, Theodore Feldman, Jose A Carvalho, Raul D Santos, Khurram Nasir

**Affiliations:** 1 Center for Prevention and Wellness Research, Baptist Health South Florida, Miami, Florida, USA; 2 Center for Research and Grants, Baptist Health South Florida, Miami, Florida, USA; 3 The Ciccarone Preventive Cardiology Center at Johns Hopkins Medical Institution, Baltimore, MD, USA; 4 Preventive Medical Center, Hospital Israelita, Sao Paolo, Brazil; 5 Lipid Institute (Incor), Sao Paolo, Brazil; 6 Robert Stempel College of Public Health and Social Work, Florida International University, Miami, Florida, USA; 7 Herbert Wertheim School of Medicine, Florida International University, Miami, Florida, USA; 8 Baptist Cardiac and Vascular Institute, Baptist Health South Florida, Miami, Florida, USA

**Keywords:** Cigarette smoking, Metabolic syndrome, Systemic inflammation, High sensitivity C-reactive protein, Cardiovascular disease risk

## Abstract

**Background:**

Emerging data suggests that the combination of smoking and metabolic syndrome (MetS) markedly increases cardiovascular disease risk well beyond that of either condition. In this study we assess if this interaction can be explained by an additive increase in the risk of systemic inflammation by MetS and cigarette smoking.

**Methods:**

We evaluated 5,503 healthy non-diabetic Brazilian subjects (mean age of 43 ± 10 years, 79% males). Participants were divided into sub-groups of smokers and non-smokers with or without MetS. High-sensitivity C reactive protein (hs-CRP) was measured to assess degree of underlying inflammation.

**Results:**

Overall (19%) had hs-CRP > 3 mg/L. In adjusted regression analyses, compared to non-smokers, there was a 0.19 mg/L (95% CI: 0.05, 0.32) increase in hs-CRP among smokers in the entire population and 0.63 mg/L (95% CI: 0.26, 1.01) increase among smokers with MetS while there was no significant increase among smokers without MetS (β = 0.09 95% CI: -0.05, 0.24). In a fully adjusted logistic regression model, smokers compared to non-smokers were 55% more likely to have elevated hs-CRP in the entire population (OR 1.55, 95% CI: 1.25, 1.92) and more than twice as likely to have elevated hs-CRP if they had MetS ( OR 2.05, 95% CI: 1.40, 3.01) while the risk was non-significant among those without MetS (OR = 1.29, 95% CI: 0.98, 1.69).

**Conclusion:**

The study demonstrates an additive effect of cigarette smoking on the risk of systemic inflammation in MetS thus highlighting the need for determining smoking status among those with MetS and aggressively targeting smoking cessation in this population.

## Introduction

Cigarette smoking and metabolic syndrome (MetS) are both well established cardiovascular disease (CVD) risk factors. With an estimated 44 million adult smokers in the United States (US) or 19% of all US adults, the health-care burden of CVD attributable to smoking is remarkable, accounting for about one-third of all CVD related deaths [1]. Globally, cigarette smoking is responsible for about 10% of all CVDs [2]. In the US, about 1 in 5 persons can be categorized as having MetS, a major public health problem likely driven by the increasing prevalence of obesity [3]. MetS is also a global health issue and is likely to persist with increasing prevalence of obesity [4,5].

Several studies have linked cigarette smoking to the presence and progression of atherosclerosis [6,7], and to markers of systemic inflammation including high-sensitivity C - Reactive Protein (hs-CRP) [8-10]. MetS is also associated with increased risk of CVD morbidity and mortality, and with all cause mortality [11]. Similar to cigarette smoking, both obesity and the MetS are associated with systemic inflammation [12]. Population studies have shown an association between cigarette smoking and the presence of MetS, and there is increasing evidence that cigarette smoking increases the risk for MetS [13-15].

Recent studies have examined the interrelationship between cigarette smoking and MetS on CVD risk [16,17]. One of these studies demonstrated a synergistic effect of cigarette smoking and MetS on CVD risk [16]. Both MetS and smoking may mediate CVD risk through shared mechanisms. Each induce systemic inflammation and the increased risk of CVD in smokers with MetS may be due, in part, to increased systemic inflammation in persons with both conditions. Though logical, there is limited evidence to support this assertion. We, therefore, tested the hypothesis that among persons with MetS, cigarette smoking worsened the risk of systemic inflammation.

## Methods

### Study population and design

This cross-sectional study was conducted in a population that consisted of 5,503 asymptomatic males and females, free from known diabetes and coronary heart disease, who presented to the Preventive Medicine Center of Albert Einstein Hospital in São Paulo, Brazil, for clinical and laboratory investigations as part of a mandatory occupational health evaluation. Each participant had clinical consultation including a history and physical examination, laboratory examination and abdominal ultrasound scanning as part of their evaluation. Information collected included demographic details, self-reported history of medical conditions such as hypertension and diabetes mellitus, use of medication including antihypertensives, antidiabetics and statins, alcohol use, and a self reported history of current cigarette smoking.

## Materials and methods

Anthropometric measurements such as weight (in kilograms), height (in meters) and waist circumference (measured at the smallest diameter between the iliac crest and the costal margin in centimeters) were obtained. Body mass index was calculated using the formula BMI = weight/(height)^2^. Blood pressure (BP) was measured using a calibrated aneroid sphygmomanometer after at least 5 minutes rest and according to guidelines from the American Heart Association [18].

Fasting blood samples were obtained for plasma lipids including high density lipoprotein cholesterol (HDL-c), low density lipoprotein cholesterol (LDL-c), triglycerides (TG), blood glucose and hs-CRP. hs-CRP levels were determined by immunonephelometry (Dade-Behring). The previously established cut-off point of >3 mg/L, a level associated with increased CVD risk in prospective studies [19] was used to define elevated hs-CRP. All tests were performed at the central laboratory of the Albert Einstein Hospital.

We defined MetS using the International Diabetes Federation criteria. This includes central obesity (waist circumference ≥ 94 cm in men or ≥80 cm in women) and any two of the following factors – hypertension, elevated triglyceride levels, reduced HDL-c, or elevated fasting glucose [20]. Smokers were defined as those who had reported smoking at least one stick of cigarette in the month prior to the evaluation while non-smokers were those who did not report smoking any cigarettes in the previous month. Based on the presence of MetS and cigarette smoking, participants were categorized into 4 groups namely - non-smokers without MetS, smokers without MetS, non-smokers with MetS and smokers with MetS. This study was approved by the local IRB and a waiver for informed consent was obtained.

### Statistical analysis

Continuous variables including hs-CRP were examined graphically for normality and are presented as mean ± SD or median (IQR). Categorical variables are expressed as percentages. Student t-tests were used to compare the means of continuous variables between smoking groups (smokers versus non-smokers) while chi-square test of independence was used to compare the frequencies of categorical variables between these two smoking groups. For comparisons of median values of hs-CRP between smokers and non-smokers the wilcoxon rank-sum test was employed.

Median hs-CRP levels as well as their interquartile ranges (IQRs) are presented for each MetS and cigarette smoking category. Similarly, the prevalence of hs-CRP for each of these groups is also graphically presented. We conducted both median linear regression and logistic regression analysis for the effect of smoking on hs-CRP, with hs-CRP expressed as continuous and categorical (hs-CRP >3 mg/L) variables respectively. We chose median linear regression because hs-CRP was non-parametrically distributed. Sub-group analyses were conducted for groups with and without MetS. Interaction terms were created and *p*-values generated. We conducted additional regression analyses assessing the effect of smoking and MetS individually and together on hs-CRP using the four subject groups described above, with non-smokers free of MetS as the reference group. Finally we conducted similar analysis in sub-groups based on their number of MetS components (elevated waist circumference, triglycerides, blood pressure and glucose, and low HDL-c as earlier mentioned). For each regression analysis univariate and multivariate analysis were conducted adjusting for age, sex, LDL-c, total cholesterol, statin use and anti-hypertensive therapy. Because the definition of MetS included hypertension, dyslipidemia and surrogates for obesity (waist circumference), they were excluded from our models. A *p* value ≤0.05 was considered statistically significant. All analysis was conducted in STATA version 12 (StataCorp. 2011. *Stata Statistical Software*: *Release 12*. College Station, TX: StataCorp LP).

## Results

### General characteristics

The study population consisted of 5,503 participants. The mean age of the population was 43.1 ± 9.4 years and about 78% of them were male. Approximately 9% (498) were self-reported current smokers while the prevalence of MetS was 20%. About 19% of the population had elevated hs-CRP (>3.0 mg/L). The median (IQR) hs-CRP in the entire population was 1.2 mg/L (0.6-1.4 mg/L) and was significantly higher in smokers than in non-smokers (1.40 mg/L vs. 1.20 mg/L p < 0.001). Among the smokers, 26% had elevated hs-CRP compared to about 19% among the non-smokers (p < 0.001). Other details of the general characteristics are available in Table 1.

**Table 1:** Population characteristics grouped according to smoking status

**Variable**	**All participants (N = 5503)**	**Non-Smokers (±SD) (N = 5,005)**	**Smokers (±SD) (N = 498)**	***p-*** **value**
**Mean age (years)**	43.48 ± 9.53	43.45 ± 9.48	43.76 ± 9.98	0.453
**Male sex (%)**	78.79	78.69	79.83	0.526
**Mean waist circumference (cm)**	91.59 ± 12.02	91.51 ± 12.02	92.40 ± 12.04	0.089
**BMI (Kg/m**^**2**^**)**	26.32 ± 3.99	26.30 ± 4.00	26.58 ± 3.72	0.100
**Metabolic syndrome (%)**	20.09	19.54	25.91	<0.001
**Mean Uric acid (mg/dL)**	5.80 ± 1.40	5.80 ± 1.40	5.90 ± 1.38	0.171
**Mean HDL-c (mg/dL)**	48.27 ± 13.06	48.48 ± 13.09	46.20 ± 12.58	<0.001
**Mean triglyceride (mg/dL)**	136.65 ± 86.69	133.87 ± 81.48	164.90 ± 122.51	<0.001
**Mean LDL-c (mg/dL)**	130.74 ± 33.25	130.65 ± 33.09	131.82 ± 34.83	0.423
**Mean total cholesterol (mg/dL)**	205.94 ± 37.12	205.60 ± 36.81	209.69 ± 39.92	0.012
**On statins (%)**	8.65	8.49	10.26	0.150
**With hepatic steatosis (%)**	36.18	35.57	42.38	0.001
**Mean fasting glucose (%)**	89.30 ± 10.26	89.27 ± 10.34	89.49 ± 10.63	0.624
**Mean SBP (mmHg) ± SD**	118.8 ± 12.7	118.7 ± 12.8	119.8 ± 12.5	0.065
**Mean DBP (mmHg) ± SD**	76.9 ± 8.1	76.9 ± 8.1	77.3 ± 8.1	0.295
**Hypertension (%)**	21.24	12.61	13.22	0.394
**On anti-hypertensive medications (%)**	12.27	12.24	12.7	0.749
**Median hs-CRP (IQR) (mg/L)**	1.2 (0.6-2.40)	1.20 (0.60-2.40)	1.40 (0.70-3.00)	<0.001
**% Elevated hs-CRP (>3.0 mg/L)**	19.24	18.60	26.10	<0.001

*SD*: standard deviation; *BMI*: body mass index; *HDL-c*: high density lipoprotein cholesterol; *LDL-c*: low density lipoprotein cholesterol; and hs-*CRP*: high sensitivity C-reactive protein.

### Relationship between smoking and MetS

The prevalence of MetS was significantly greater among smokers than non-smokers (26.5% vs. 19.8% p < 0.001). In logistic regression analysis before and after controlling for age and sex, antihypertensive and antidiabetic medications and total cholesterol, the odds of MetS was 46% and 36% greater among smokers compared to non-smokers (Unadjusted OR 1.46 95% CI: 1.18, 1.81 p <0.001; Adjusted OR 1.36 95% CI: 1.08, 1.72 p = 0.008).

### Relationship between smoking and hs-CRP among persons with and without metabolic syndrome

**Figure 1:**
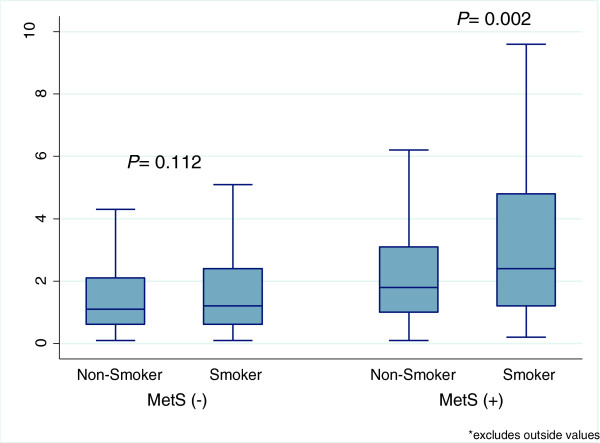
**Box plots comparing median and interquartile ranges of hs**-**CRP between smokers and non**-**smokers in populations with and without metabolic syndrome (MetS).**

Among those without MetS, there was no significant difference in the median hs-CRP between smokers and non-smokers (*p* = 0.11) however, among those with MetS, the median hs-CRP was significantly higher in smokers (2.4 mg/L, IQR 1.2-4.8 mg/L) than in non-smokers (1.8 mg/L, IQR 1.0-3.1 mg/L) (*p* = 0.002; Figure 1). In the unadjusted linear regression analysis comparing smokers to non-smokers, smoking was associated with a 0.20 mg/L (95% CI: 0.07, 0.33) increase in median hs-CRP in the entire population and 0.60 mg/L (95% CI: 0.10, 1.01) increase in hs-CRP among those with MetS (Table 2). However, among those without MetS, smoking had no effect on hs-CRP (regression coefficient 0.10, 95% CI: -0.04, 0.24). Similar associations were found in the fully adjusted model. In addition, there was significant interaction between MetS and smoking in univariate (p = 0.002) and both multivariate analyses (p = 0.001) (Table 2).

**Table 2:** **Linear and logistic regression analyses for the effect of cigarette smoking on Hs**-**CRP and interaction with metabolic syndrome**

**Median linear regression coefficients (95% CI)**				
	**Total**	**MetS absent**	**MetS present**	***P *** **interaction**
**Model 1**	0.20 (0.07, 0.33)	0.10(-0.04, 0.24)	0.60 (0.23, 0.97)	0.002
**Model 2**	0.18 (0.04, 0.32)	0.11 (-0.04, 0.26)	0.60 (0.10, 1.01)	0.001
**Model 3**	0.19 (0.05, 0.32)	0.09 (-0.05, 0.24)	0.63 (0.26, 1.01)	0.001
**Logistic regression odds ratio for elevated hs-CRP (95% CI)**				
	**Total**	**MetS absent**	**MetS present**	***P *** **interaction**
**Model 1**	1.54 (1.25, 1.91)	1.27 (0.97, 1.66)	2.02 (1.39, 2.93)	0.049
**Model 2**	1.55 (1.26, 1.93)	1.28 (0.97, 1.67)	2.05 (1.40, 2.99)	0.05
**Model 3**	1.55 (1.25, 1.92)	1.29 (0.98, 1.69)	2.05 (1.40, 3.01)	0.076

**Model 1**: unadjusted; **Model 2**: adjusted for age and sex; and **Model 3**: adjusted for age, sex, LDL, total cholesterol, statins and anti-hypertensive medications hs-CRP: high sensitivity C-reactive protein; and MetS: metabolic syndrome elevated hs-CRP = hs-CRP >3 mg/L.

**Figure 2:**
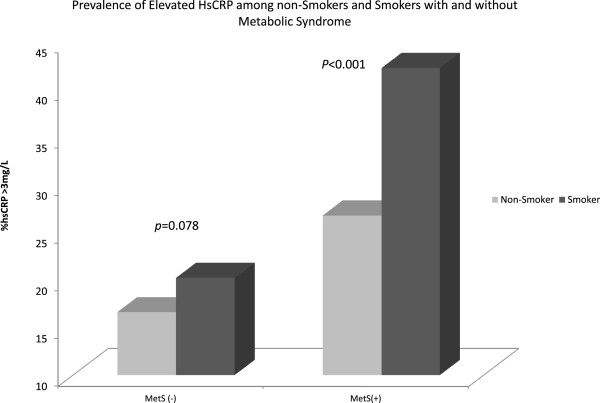
**Prevalence of elevated hs**-**CRP among smokers compared to non**-**smokers in populations with and without metabolic syndrome.**

In the group without MetS, there was no significant difference in the prevalence of elevated hs-CRP among smokers versus non-smokers (20% vs. 17% p = 0.08) however among those with MetS the hs-CRP prevalence was 42.4% among smokers compared to 26.8% among non-smokers (p < 0.001).These results are depicted in Figure 2. In a fully adjusted logistic regression model, cigarette smoking was associated with a 55% increase in the odds of elevated hs-CRP in the entire population (OR 1.55, 95% CI: 1.25, 1.92) and more than double the odds of elevated hs-CRP among those with MetS (OR 2.05, 95% CI: 1.40, 3.01). However, there was no significant effect of smoking on hs-CRP in the population without MetS (OR 1.29, 95% CI: 0.98, 1.69). Again, there was significant interaction between MetS and smoking on hs-CRP in unadjusted (p = 0.049) and the age and sex adjusted models (*p* interaction = 0.05) but not in the fully adjusted model (*p* interaction = 0.076). Details of the results of the logistic regression analysis are shown in Table 2.

### Comparison of hs-CRP among non-smokers without Mets to smokers with MetS, smokers without MetS and non-smokers with MetS

There was a stepwise increase in the median hs-CRP and the prevalence of elevated hs-CRP in the following order: non-smokers without MetS, smokers without MetS, non-smokers with MetS and smokers with MetS (Figures 1 and 2). In adjusted regression analysis hs-CRP was 1.3 mg/L (95% CI: 1.0, 1.6) and 0.7 mg/L (95% CI: 0.6, 0.8) higher among smokers with MetS and non-smokers with MetS compared to non-smokers without MetS. However, there was no significant increase in hs-CRP associated with smokers without MetS compared with non-smokers without MetS (β = 0.11 95% CI: -0.1, 0.3). Again, compared with non-smokers without MetS, smokers with MetS and non-smokers with Mets were 4 times and 2 times more likely to have elevated hs-CRP while the risk was not significantly elevated for those who smoke but do not have MetS (OR 1.30 95% CI: 1.0, 1.7). Details can be found in Table 3.

**Table 3:** Linear and logistic regression analyses for the combined effect of cigarette smoking and metabolic syndrome on hs-CRP

**Median linear regression coefficients (95% CI)**			
	**Smokers with no MetS**	**Non**-**smokers with MetS**	**Smokers with MetS**
**Model 1**	0.10 (-0.06, 0.26)	0.70 (0.60, 0.80)	1.3 (1.06, 1.56)
**Model 2**	0.12 (-0.04, 0.29)	0.73 (0.62, 0.84)	1.39 (1.13, 1.66)
**Model 3**	0.11 (-0.05, 0.27)	0.69 (0.58, 0.79)	1.29 (1.03, 1.55)
**Logistic regression odds ratio for elevated hs-CRP (95% CI)**			
	**Smokers with no MetS**	**Non**-**smokers with MetS**	**Smokers with MetS**
**Model 1**	1.27 (0.97, 1.66)	1.83 (1.56, 2.16)	3.70 (2.59, 5.28)
**Model 2**	1.28 (0.97, 1.67)	2.09 (1.76, 2.48)	4.24 (2.95, 6.08)
**Model 3**	1.30 (0.99, 1.71)	2.02 (1.69, 2.41)	4.00 (2.77, 5.08)

**Model 1**: unadjusted; **Model 2**: adjusted for age and sex; and **Model 3**: adjusted for age, sex, LDL, total cholesterol, statins and anti-hypertensive medications Hs-CRP: high sensitivity C-reactive protein; and MetS: metabolic syndrome elevated hs-CRP = hs-CRP >3 mg/L.

## Discussion

In this study population, smoking was associated with prevalent MetS and in those who had MetS, smoking more than doubled the risk of systemic inflammation. The combination of smoking and MetS was also associated with a four-fold increase in the likelihood of systemic inflammation when compared to non-smokers without MetS. Interestingly, smokers without MetS did not have elevated risk of systemic inflammation. Taken together, our results imply that smoking significantly and adversely modifies the effect of MetS on systemic inflammation, supporting our earlier hypothesis that smoking worsens the systemic inflammation among persons with MetS. We were however surprised that there was no significant effect of smoking on systemic inflammation among persons who did not have MetS.

Several studies have demonstrated an association between smoking and MetS and this association has been summarized in a recent review and meta-analysis [15]. Smoking has a well documented, independent, dose–response relationship with systemic inflammation as measured by hs-CRP [21-23]. Consistent with prior reports, in the present study smoking was associated with both MetS and systemic inflammation. One of the arguments behind the development of MetS in smokers is the induction of insulin resistance [24-26], the major driver of the MetS. Our study shows that cigarette smoking further escalates systemic inflammation in persons with MetS, as shown by an additional increase of 0.6 mg/L in the level of hs-CRP and a 100% increase in the risk of elevated hs-CRP (>3 mg/L). Elevated hs-CRP has been closely linked with the development of atherosclerosis and is associated with the development of and mortality from CVD [27]. Though not proven here, our study suggests that aggravation of systemic inflammation by cigarette smoking may account for the increased risk of CVD in cigarette smokers with MetS.

The major strength of our study is that it was carried out in a large cohort of over 5,500 individuals free of known CVD and with information regarding important covariates. The analysis is also unique as it is one of the earliest to demonstrate an additive effect of cigarette smoking on the risk of systemic inflammation in persons with MetS. However, we are limited by several factors. The cross-sectional design precludes defining the temporal relationship between smoking and hs-CRP. The study population comprised mainly of men mostly in their forties and free of CVD. Therefore, the findings cannot be generalized to women or populations in a different age bracket. The fact that most of the covariates, including the primary exposure – cigarette smoking is self-reported opens up the possibility of misclassification of exposure and the potential for a misclassification bias. Smokers were not classified as never smokers or non smokers, which would have reduced the bias of “sick quitter effect”. In addition, no measure of smoking burden was taken into account, all of which may have affected our results and may account for lack of a significant difference in hs-CRP among those without MetS.

### Implications and conclusions

Both smoking and MetS are among the leading causes of preventable CVD related deaths in the US and globally. Although the prevalence of smoking and other traditional CVD risk factors (dyslipidemia and hypertension) in the US has decreased from 1960 to 2000, the same period has seen a dramatic rise in the prevalence of obesity and MetS [1,28]. Despite the decline in smoking rates, 19% of the US population are smokers and cigarette smoking still accounts for over 400,000 deaths yearly in the US, 32% of which are CVD related [1,29]. Globally, tobacco use accounts for about 10% of all CVD related mortality, with the highest occurrences being in low to mid-income countries [2]. Yet awareness about the cardiovascular implications of cigarette smoking are still unacceptably low considering that smoking is a completely preventable cause of CVD [2].

From the results of this study, the absence of smoking, even in the presence of MetS is associated with a 50% reduction in risk of systemic inflammation. Other studies have demonstrated that smoking cessation substantially reduces CVD risk [30]. This study also emphasizes the importance of assessing smokers for metabolic abnormalities and establishing smoking status in those who present with features of the MetS. Smoking cessation attempts need to be more rigorous in those with MetS. There is strong argument that smoking cessation may benefit smokers with MetS [17] since smoking cessation improves insulin sensitivity [31] and could break the interaction between the two.

Our study, as well as others, underscores the need for active pursuant of smoking cessation in the general population by modifying smoking related public health policies and intensive interventions in clinical practice especially among those who have metabolic abnormalities or MetS. Moreover, further studies are needed to understand the processes responsible for the additive effect of smoking and MetS on inflammation.

## Abbreviations

BMI: Body mass index; CVD: Cardiovascular disease; HDL-c: High density lipoprotein cholesterol; hs-CRP: High sensitivity C reactive protein; LDL-c: Low density lipoprotein cholesterol; MetS: Metabolic syndrome; US: United States of America.

## Competing interests

The authors declare that they have no competing interests.

## Authors’ contributions

Conceived and designed the study: JA, AF, SN. Performed the experiments: JC, VC, SN. Analyzed the data: JA, VN. Wrote the paper: JA, MS, AP, N. Reviewed the manuscript for content and scientific accuracy: AA, FV, BB, SA P, MC, o VC SN. All authors read and approved the final manuscript.
